# A comparative study on the cardiac morphology and vertical jump height of adolescent black South African male and female amateur competitive footballers

**DOI:** 10.5830/CVJA-2017-032

**Published:** 2018

**Authors:** Jean-Luc Gradidge Philippe, Constantinou Demitri

**Affiliations:** Centre for Exercise Science and Sports Medicine(CESSM), Faculty of Health Sciences, University of the Witwatersrand, Johannesburg, South Africa; Centre for Exercise Science and Sports Medicine(CESSM), Faculty of Health Sciences, University of the Witwatersrand, Johannesburg, South Africa

**Keywords:** adolescent, black South African, footballers, cardiac morphology, vertical jump height

## Abstract

**Objective:**

The aim of this comparative study was to determine the gender differences in cardiac morphology and performance in adolescent black South African footballers.

**Methods:**

Anthropometry, electrocardiography and echocardiography data were measured in 167 (85 males and 82 females) adolescent black South African footballers (mean age: 14.8 ± 1.3 years). Vertical jump height was used as a performance measure of explosive lower –limb power.

**Results:**

The males had less body fat compared with the females (12.1 ± 3.6 vs 16.8 ± 4.1%, p < 0.05), while females had higher left ventricular end –diastolic diameters compared with males (48.7 ± 3.7 vs 40.7 ± 8.1, p < 0.05). Vertical jump height was higher in males (37.2 ± 10.3) compared with females (31.2 ± 8) and was inversely associated with body fat (â = –0.2, p < 0.05) and positively associated with lean mass (â = 0.5, p < 0.05).

**Conclusion:**

The findings showed that adolescent black South African male footballers had a performance advantage over females for explosive lower –limb power, which was explained by differences in body composition and not cardiac morphology.

Football is a popular sport, particularly among adolescents in the developing world.^1^ Adaptive changes to the heart are assumed with chronic participation in football,^2^ however little is known of the cardiac morphology of African adolescents who participate in this game.^3^ Hypertrophy of the cardiac muscle is the main consequence of training, contributing to the higher level of performance compared with the sedentary, age –matched, non –football population.^4^

Although such cardiac adaptations provide clear physiological advantages for the ability to withstand play during the intermittent nature of football, it is important to distinguish between the physiological and pathological heart. Echocardiography and electrocardiography are therefore important investigative tools in football players, first to screen for and monitor at –risk players, and second to monitor physiological development related to football training.^5^

Lower –limb dynamic functionality, particularly explosive power during sprinting and jumping activities, is a key characteristic of football.^6^ These contribute to the speed and strength demands of football performance movements.^7^ It has been reported that adolescent participants with congenital heart disease produced lower peak jump heights compared with age –matched ‘normal’ controls.^6^ The converse is unknown, namely whether optimal performance in these activities may depend on the structural cardiac adaptations that follow football training.

The aims of this comparative study were: (1) to determine the gender differences in cardiac morphology and performance in competitive adolescent South African footballers; and (2) to determine the factors associated with explosive jump height and measures of cardiac morphology in these footballers.

## Methods

Data for this comparative study of adolescent black South African footballers were collected from seven of the nine provinces (the Eastern Cape, Free State, Gauteng, KwaZulu – Natal, Limpopo, Mpumalanga and Western Cape). Participants included were within the age range of 12 to 18 years, without injury and actively involved in competitive amateur –level football. Ethical clearance for the study was granted by the University of the Witwatersrand (M140513).

Participants dressed with minimal clothing on the testing day. No shoes were worn for the anthropometric measurements. Height (m) was measured using a stadiometer (Seca 217, UK) and weight (kg) was measured using a digital scale (Seca 844, UK). Body mass index (BMI) was calculated as weight (kg)/ height (m^2^) and presented using BMI for age guidelines.^8^

The Omron sphygmomanometer (Canada) and accompanying stethoscope were used to measure systolic and diastolic blood pressure (BP) with the participant in a seated position after five minutes’ rest period. Three measurements were taken and the average of the second and third BP measurements was recorded. Skinfold measurements were used to determine proxy measures of body fat and muscle mass using standardised methods.^9^

Echocardiography was performed and measures of cardiac morphology included interventricular septal (IVS) thickness, ejection fraction percentage and left ventricular end –diastolic diameter (LVED) [Mindray DP –6600, Shenzhen, China (using a 2.5–3.5 MHz cardiac transducer)]. Resting and stress electrocardiograms (ECG) were recorded using a Schiller AT 6 ECG machine (Schiller AG, Switzerland) using standardised treadmill protocols.^9^ Vertical jump height (VJH) was measured as the maximal jump height from a two –legged standing starting position. The best result from three trials was recorded.^10^ The sit –and –reach test was used to measure truncal flexibility (cm).^9^

## Statistical analysis

Statistica 13.2 (StatSoft, Tulsa, USA) was used for all analyses. The Student’s t –test was used to compare anthropometric, cardiac and fitness profiles between male and female participants (p < 0.05). Complete data were not available for all measures as more advanced body composition measuring tools, echocardiography and instruments used for performance were available at only selected regions. The mean of the sample was used for participants who had missing data.

To determine whether the sample size was suitable, a null hypothesis of < 5% was rejected. Assuming a standardised difference of 0.58 with 90% power for LVED, the minimum number of participants required in each group is 78 with the alpha value set at 5%.11 The actual sample size was above the minimum required.

Bivariate analyses were conducted to determine the correlation between variables using Pearson’s correlation by gender. Multivariable linear regression analyses were conducted to determine whether independent variables were associated with the explosive power outcome (VJH) and the cardiac morphology outcomes (ejection fraction percentage, IVS thickness, and LVED. Based on the outcome of the bivariate analysis, the following independent variables were included in the initial multiple regression model for the fitness outcome: age, gender, systolic BP, diastolic BP, body fat, lean muscle mass, IVS thickness, ejection fraction and LVED.

The following independent variables were included in the initial multivariable linear regression models for the three cardiac outcomes: age, gender, systolic BP, diastolic BP, body fat, muscle mass, resting heart rate, IVS thickness, ejection fraction, LVED and VJH. Multico –linearity was checked using variance inflation factor (VIF) analysis. All independent variables had VIFs < 2.2, indicating no co –linearity.

## Results

The participants were from Gauteng (n = 35), Kwa –Zulu Natal (n = 27), Mpumalanga (n = 36), Western Cape (n = 15), Eastern Cape (n = 19), Free State (n = 19) and Limpopo (n = 16). The mean age of the study population was 14.8 ± 1.3 years, with a mean BMI of 20.6 ± 2.4 kg/m^2^. The female (n = 82) footballers were younger compared with the males (n = 85), and presented with significantly higher body fat measures and lower lean mass (Table 1).

**Table 1:** Characteristics of adolescent South African footballers by gender

*Variables*	*Total sample (n = 167)*	*Males (n = 85)*	*Females (n = 82)*	*Percentage difference†*
Proportion (%)	100	50.9	49.1	1.8
Age (years)	14.8 ± 1.3	15.5 ± 1.1	14.1 ± 1.1^*^	9.5
Height (m)	1.61 ± 0.1	1.66 ± 0.1	1.56 ± 0.1^*^	6.2
Weight (kg)	54 ± 8.5	58.3 ± 7.2	49.5 ± 7.3^*^	16.3
BMI (kg/m2) by age range
12–13 years (n)	19.9 ± 2.3 (26)	20.1 ± 1 (4)	19.8 ± 2.5 (22)	1.5
14–15 years (n)	20.7 ± 2.1 (92)	20.9 ± 1.4 (35)	20.5 ± 2.5 (57)	1.9
16–17 years (n)	21.1 ± 2.2 (49)	21.1 ± 1.9 (46)	20.7 ± 0.2 (3)	1.9
Body fat (kg)	14.4 ± 4.5	12.1 ± 3.6	16.8 ± 4.1^*^	32.5
Lean mass (kg)	51.1 ± 4.9	53.5 ± 3.9	48.7 ± 4.7^*^	9.4
Ejection fraction (%)	64 ± 7.2	64.8 ± 7	63.1 ± 7.3	2.7
IVS thickness (mm)	9.2 ± 1.7	9.9 ± 1.5	8.4 ± 1.6^*^	16.4
LVED	44.8 ± 7.5	48.7 ± 3.8	40.7 ± 8.1^*^	17.9
Resting heart rate (bpm)	68.9 ± 9.8	65.8 ± 10.6	72.2 ± 7.6^*^	9.3
Peak heart rate (bpm)	174.8 ± 9	176.7 ± 8.1	172.8 ± 4.8^*^	2.2
Systolic BP (mmHg)	113.8 ± 10	117.2 ± 10.9	110.3 ± 7.5^*^	6.1
Diastolic BP (mmHg)	71.2 ± 8.2	72.3 ± 8.1	69.3 ± 8^*^	4.2
VJH (cm)	37.2 ± 10.3	43.1 ± 8.9	31.2 ± 8^*^	32
Trunk flexibility (cm)	40.3 ± 4.9	40.3 ± 3.7	40.4 ± 5.9	0.3

Data presented as mean ± SD; ^*^p < 0.05 versus males; †Percentage difference (male minus female/male).

BMI: body mass index; BP: blood pressure; IVS: interventricular septal thickness;

VJH: vertical jump height; LVED: left ventricular end-diastolic diameter.

Resting BP, IVS thickness and LVED were significantly higher in males compared with females; however end –diastolic volumes were similar. Flexibility did not differ between the males and females, however VJH was significantly higher in males compared with females (Table 2).

**Table 2:** Pearson’s correlations for VJH, ejection fraction, IVS thickness and LVED presented by gender

	*VJH*		*Ejection fraction*		*IVS thickness*		*LVED*	
*Variables*	*Males*	*Females*	*Males*	*Females*	*Males*	*Females*	*Males*	*Females*
Age	0.4^*^	0.1	0.2	0.01	–0.2	–0.1	–0.2	–0.1
BMI	0.2	–0.1	–0.2	0.1	0.2	–0.1	0.2^*^	–0.1
Body fat	–0.3^*^	–0.4^*^	–0.5^*^	0.1	0.4^*^	–0.2	–0.1	–0.2
Lean mass	0.7^*^	0.7^*^	0.1	-0.1	-0.1	–0.3^*^	0.1	–0.3^*^
Ejection fraction	0.2	–0.1			–0.4	0.5^*^	–0.02	0.5^*^
IVS thickness	–0.1	–0.2	–0.4^*^	0.5^*^			0.1	0.9^*^
LVED	0.1	–0.2	–0.02	0.5^*^	0.1	0.9^*^		
Resting heart rate	–0.2	–0.3^*^	0.3^*^	0.1	–0.4^*^	–0.3^*^	–0.1	–0.3^*^
Systolic BP	–0.1	0.2	0.1	–0.004	0.1	–0.02	0.03	–0.04
Diastolic BP	–0.3^*^	0.2	–0.1	–0.1	0.04	–0.5^*^	–0.1	–0.6^*^
VJH			0.2	–0.1	–0.1	–0.2	0.1	–0.2
Trunk Flexibility	–0.3^*^	0.02	0.1	–0.04	–0.2^*^	0.1	0.1	0.2

Data presented as r coefficient for males (n = 85) and females (n = 82); ^*^p < 0.05. BMI: body mass index; BP: blood pressure; LVED: left ventricular end-diastolic diameter; IVS: interventricular septal thickness; VJH: vertical jump height.

For those who had cardiac auscultation conducted (79 males and 20 females), none of the females presented with abnormal auscultation, while five of the males had functional systolic ejection murmurs (6.3%) and one had a tricuspid regurgitation murmur (3/6) (1.3%). No resting or stress ECGs showed any pathological abnormalities.

Using bivariate analysis, there was a significant positive correlation between VJH and age, and lean mass, while body fat, diastolic BP and trunk flexibility were negatively correlated in males. In females, body fat and resting heart rate were negatively correlated with VJH, while lean mass was positively correlated.

In males, body fat and IVS thickness correlated negatively with ejection fraction, while resting heart rate correlated positively. In females, IVS thickness and LVED correlated positively with ejection fraction. In males, IVS thickness was positively associated with body fat, and negatively associated with ejection fraction, resting heart rate and flexibility. In females, IVS thickness was negatively associated with lean mass, ejection fraction, resting heart rate and diastolic BP, while positively associated with LVED. In males, LVED was positively associated with BMI. In females, LVED was positively associated with ejection fraction, resting heart rate and IVS thickness, while negatively associated with diastolic BP and lean mass.

Table 3 displays the associations for the fitness and cardiac morphology outcomes of the study population. All models were adjusted for age and gender. The multivariable regression models explained 66, 19, 59 and 67% of the variation in VJH, ejection fraction, IVS thickness and LVED, respectively. VJH was positively associated with lean mass, and negatively associated with body fat. Ejection fraction was positively associated with LVED and resting heart rate. Resting heart rate was inversely associated with IVS thickness, while body fat and LVED were positively associated. LVED was positively associated with ejection fraction, body fat and IVS thickness, while negatively associated with diastolic BP.

**Table 3:** Multivariable linear regression models for determining the influence of body composition, blood pressure, cardiac morphology and performance on VJH, LVED, IVS thickness and ejection fraction

*Exposure*	*VJH*	*Ejection fraction (%)*	*IVS Thickness*	*LVED*
Body fat	–0.2^*^	–0.1	0.2^*^	0.2^*^
Ejection fraction	0.04		–0.02	0.2^*^
Lean mass	0.5^*^	0.04	–0.1	–0.1
IVS thickness	0.004	–0.04		0.6^*^
LVED	–0.03	0.5^*^	0.7^*^	
RHR	–0.1	0.3^*^	–0.2^*^	–0.001
Systolic BP	0.1	0.1	0.02	0.1
Diastolic BP	-0.02	-0.04	0.02	–0.2^*^
VJH		0.1	0.01	–0.03
R^2^	0.66^*^	0.19^*^	0.59^*^	0.67^*^

Data presented as adjusted β; ^*^p < 0.05. All models were adjusted for age and gender. LVED: left ventricular end-diastolic diameter; IVS: interventricular septal thickness; VJH: vertical jump height; RHR: resting heart rate.

## Discussion

Football is the most popular sport globally, with adolescents making up a large proportion of participants. In most sports that predominantly utilise the aerobic system, regular participation provides significant cardiovascular adaptations as an adaptation to improve performance.^2^ Our study findings primarily add to the limited body of evidence on the cardiac morphology of adolescent African footballers.

The findings also show the gender differences in physiological profile and selected performance outcomes, and that selected cardiac parameters were not associated with dynamic VJH. Males were taller and weighed more than the female study participants, which is likely due to the higher lean muscle mass observed in the male participants.^12^ These body composition differences are expected in adolescents of this age, but little is known about the differences in cardiac morphology of African populations.

The higher lower –limb dynamic explosive power values observed in male compared with female footballers supports the well –known notion of gender functional strength differences. In this study population, however, the sociopolitical landscape of South Africa cannot be excluded as an additional source of this discrepancy.^13^

Despite encouraging participation in sport, policy makers fail to account for the limited resources and lack of accessibility experienced by female athletes in South Africa. There are still barriers even when there are opportunities. For example, most premier league football teams have the capability to allow for the development of adolescent footballers, but the training is often performed in the late afternoons to night –time. With personal safety being a concern, these facilities may not easily be accessible for adolescent female footballers, even if located within walking distance. Therefore female subjects may not fully engage in football training as a consequence.

The daily demands of schoolwork and other life stresses can further de–emphasise the central focus on football. In addition, incentives to participate in professional football are currently more favourable for male subjects compared with females; however, gender equality in various sports is being addressed.

Although studies have shown that the untrained female can improve cardiovascular function by participating in recreational competitive football,^14^ males still have a more pronounced physiological advantage over age –matched females. The diameters of the male participants’ left ventricles are similar to those of footballers of African descent living in Europe,^15^ suggesting some degree of genetic heritability.

On the other hand, the female participants seem to be more aligned with the adaptations experienced by volleyball athletes.^16^ This is evident by the finding that females have smaller heart sizes and lower left ventricular mass compared with males.^17^ Therefore, even though the cardiovascular adaptations to aerobic sport are similar, the absolute difference in cardiac structure is higher in male subjects. Moreover, the variation in cardiac morphology can also be explained by the fact that height is highly associated with heart size and function,^17^ and male subjects in our study were taller compared with females.

The vertical jump test is not only an indicator of explosive strength, but also of neuromuscular adaptation. Our study findings show that age, gender and body composition have an influence on the difference in results for this variable. Therefore, the lower VJH values in females can be explained by lower muscle mass, younger age and increased body fat.

It is worth considering the specificity of training to explain the advantage noted in males. Current knowledge shows that for optimal neuromuscular adaptation, athletes need to engage in a progressive strength –training programme, and actively challenge the neuromuscular system.^18^ Tendons assist with functional movement by acting as shock absorbers and energy capacitors within the muscle–tendon complex.^19^ Further research is needed to determine whether female footballers have lower jump height as a result of lower tendon compliance for explosive activities.

The findings of this study highlight the interconnected characteristic of the various cardiac muscle components. This points to the complexity involved in trying to comprehend cardiac development in footballers. For example, our study findings show that lean muscle mass was associated with LVED volume in the regression analyses, but football training is seasonal, with the pre –season representing a period of strength development, while the competitive phase of the sport is typical of peak performance during match play. This suggests that the training adaptation is non –linear, but rather fluctuates depending on external exercise demand.

The level of primary sex hormones also alters during the football seasons and depends on internal (e.g. menstrual cycle in females) and external stresses (e.g. more resistance training in males, often to enhance body image). Serum testosterone levels can influence muscle architecture and function, and it is during times of peak training stress when the advantages of this anabolic hormone are most notable. In football, this usually occurs during the aerobic component of play, when the slow –twitch muscle fibres are most active. The body adapts by improving cardiac output, essentially by increasing left ventricular mass,^2^ which is more pronounced in male athletes.

The main limitation of this study is that causality could not be inferred in this comparative study, indicating the need for longitudinal studies of adolescent African footballers to confirm our findings.

## Conclusion

This comparative study demonstrated the gender differences in performance as a result of physiological and cardiovascular advantages in male subjects. In addition, football training can remodel body composition, resulting in enhanced jumping ability, which is essential during competitive match play.
